# Nanoimprinted multifunctional nanoprobes for a homogeneous immunoassay in a top-down fabrication approach

**DOI:** 10.1038/s41598-021-85524-8

**Published:** 2021-03-16

**Authors:** Hubert Brueckl, Astrit Shoshi, Stefan Schrittwieser, Barbara Schmid, Pia Schneeweiss, Tina Mitteramskogler, Michael J. Haslinger, Michael Muehlberger, Joerg Schotter

**Affiliations:** 1 Department for Integrated Sensor Systems, Danube University Krems, Wiener Neustadt, Austria; 2 PROFACTOR GmbH, Steyr-Gleink, Austria; 3 AIT Austrian Institute of Technology, Vienna, Austria

**Keywords:** Diagnostic devices, Nanofabrication and nanopatterning, Magnetic properties and materials, Biosensors, Nanoparticles, Sensors and probes

## Abstract

Multifunctional nanoparticles are discussed as versatile probes for homogeneous immunoassays for in-vitro diagnostics. Top-down fabrication allows to combine and tailor magnetic and plasmonic anisotropic properties. The combination of nanoimprint lithography, thin film deposition, and lift-off processing provides a top-down fabrication platform, which is both flexible and reliable. Here, we discuss the material compositions and geometrical designs of monodisperse multicomponent nanoparticles and their consequences on optical and magnetic properties. The rotational hydrodynamics of nanoparticles is measured and considered under the influence of magnetic shape anisotropy in the framework of the Stoner-Wohlfarth theory. The plasmon-optical properties are explained by discrete-dipole finite-element simulations. Rotational dynamical measurements of imprinted nanoprobes for two test proteins demonstrate the applicability as highly sensitive biomolecular nanoprobes.

## Introduction

The ability to agitate and manipulate magnetic nanoparticles by an external magnetic field has triggered a lot of research for clinical applications^1–3^. Their high potential as contrast enhancement agents for magnetic resonance imaging^4–6^, therapeutic drug and gene delivery^7,8^, magnetic separation of cells^9,10^, in-vivo cancer diagnostics^11^ or for hyperthermia cancer treatment using radio frequency methods^12–14^ has already been reported. The transformation of magnetic nanoparticles into nanoprobes by a specific bio-functionalization is key in many heterogeneous and homogeneous biosensor approaches, which enables the detection of biological entities such as disease-related biomarkers, DNA or proteins^15–18^. Heterogeneous biosensor assays such as enzyme-linked immunosorbent assays (ELISA) are standard diagnostic tools, which show a high sensitivity and wide dynamic range^19^. However, intrinsic problems, e.g., steric hindrance^20^, restricted diffusion^21^ and extensive sample preparations, limit their applicability for point-of-care testing^22^. These issues can be circumvented in homogeneous immunoassay principles, where the signal generating nanoprobes are mixed with the analyte sample and the following target molecule detection is carried out directly within the probe-analyte mixture.

The homogeneous sensing principle of this work relies on changes in the rotational dynamics of optically and magnetically anisotropic nanoparticles^23^. The feasibility and potential of this nanoparticle-based homogeneous immunodiagnostics for simple, fast and sensitive biomarker detection has been demonstrated using chemically synthesized nanorods^24,25^. Although the chemically synthesized core–shell nanoprobes were of high quality, they still show a relevant distribution in size, which leads to a spread in the measured signal and loss in sensitivity. Critical limiting factors inherent to the bottom-up chemical fabrication methodology are thermodynamic instability and nucleation formation resulting in uncontrolled shape, size, structure variations and inhomogeneous shell layers^26,27^. Moreover, no noticeable and reliable plasmonic excitation could be observed in these chemically synthesized core–shell nanoparticles, which would significantly increase the sensitivity^23^.

The challenges inherent in chemical synthesis may be overcome by using “top-down” physical fabrication methods such as nanoimprint lithography (NIL). NIL represents an inexpensive technique to replicate high resolution (< 15 nm) nanostructures on a large scale using a single imprint step^28,29^. By combining NIL with thin film deposition, nanoparticles of almost arbitrary shape (round, elliptical, rectangular etc.) and material composition are producible^30^. This enables the fabrication of nanoparticle with tailored magnetic and plasmonic properties by selecting appropriate layers from a broad range of well-defined homogenous noble metals, proper magnetic materials, and oxides^31^. Kwon et al.^32^ already demonstrated the feasibility to fabricate large amounts of highly uniform sombrero-shaped magnetite nanoparticles using NIL.

In this study, we combine NIL, thin film technology, and lift-off processing to engineer monodisperse magneto-plasmonic nanoparticles of high quality and reproducibility. The magnetic, plasmon-optical and hydrodynamic properties of the multicomponent nanoparticles are investigated and compared to numerical simulations. In the last chapter, their suitability for biomolecular detection is validated by two test proteins.

## Results and discussion

### Detection scheme of a homogeneous biosensor with optically and magnetically anisotropic nanoparticles

**Figure 1:**
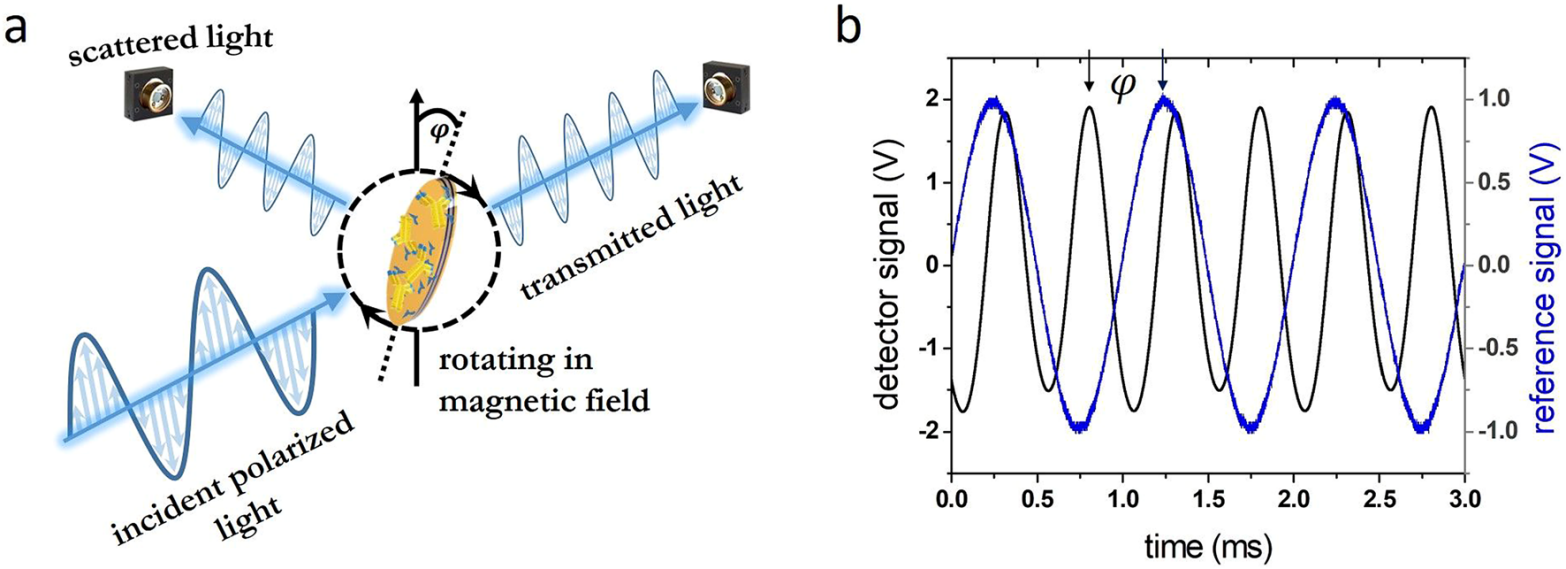
(**a**) Schematic illustration of the detection principle: The functionalized nanoparticle follows a rotating magnetic field, but its orientation lags behind by an angle φ. This angle increases with the number of bound molecules. Consequently, the measured phase lag is proportional to the target molecule concentration in the sample solution. (**b**) Frequency-doubled detector signal (black) and reference coil signal (blue) with phase lag φ.

The detection principle of the presented homogeneous biosensor is based on the optical observation of changes in the rotational dynamics of both magnetically and optically anisotropic nanoparticles (Fig. 1a). Details of the measurement setup are described in^33^. The nanoparticles are functionalized with complementary receptors and mixed with the analyte sample such as serum or whole blood. An external time-varying magnetic field $\overrightarrow{H}$ is utilized to rotate the nanoprobes in the (x,y)-plane perpendicular to the incident light (Fig. 1). The particles specifically bind target molecules on their surface, which leads to an increase of their hydrodynamic volume. Figure 1b exemplary shows the characteristic frequency-doubled detector raw signal recorded in transmission geometry for a nanoparticle concentration of 256 µg/ml immersed in ultra-pure water. The reference is the driving field of the magnetic coils. In equilibrium, the magnetic torque $\overrightarrow{M}\times \overrightarrow{B}$ ($\overrightarrow{M}$: nanoparticle magnetization; $\overrightarrow{B}={\mu }_{0}\overrightarrow{H}$: magnetic induction induced in the sample by the applied magnetic field H) on the nanoparticles is balanced by the rotational viscous torque, which is directly proportional to the fluid viscosity $\eta$, the hydrodynamic volume ${V}_{hydro}$ and the angular velocity $\overrightarrow{\omega }$^34^:

1 $$\overrightarrow{M}\times \overrightarrow{B}-6 \eta \bullet {V}_{hydro}\bullet \overrightarrow{\omega }=0$$

For a field rotating in the (x,y)-plane, the nanoparticles also rotate in this plane with the same speed as the field. The nanoparticles, however, experience a drag torque resulting in an increased viscous phase lag α between the magnetization $\overrightarrow{M}=\left(M\mathrm{cos}\left(\omega t-\alpha \right),M\mathrm{sin}\left(\omega t-\alpha \right),0\right)$ and the external driving magnetic field $\overrightarrow{H}=\left(H\mathrm{cos}\left(\omega t\right),H\mathrm{sin}\left(\omega t\right),0\right)$.

As shown in^35^, the nanoparticle rotation exhibits two distinct modes of motion: synchronous and asynchronous rotation. Synchronous rotation occurs for low ω values because the magnetic and viscous torques are in equilibrium and the phase difference between the particle and the magnetic field is constant. At a critical frequency, the phase delay becomes equal to π/2 and the particle rotation becomes asynchronous: the nanoparticle undergoes a periodic sequence of forward and backward turns, rotating, on average, in the same direction as the field but with a frequency lower than ω. The biomolecular detection operates only in the synchronous regime. Although the frequency is below the critical value, another instability may occur, which is not mentioned in^35^ and described next.

For nanoparticles in an aqueous sample solution at temperatures around room temperature, another important addition to the torque balance described in Eq. (1) is the Brownian motion of the nanoparticles. When including this, the viscous phase lag α ^36^ for rigid magnetic dipole nanoparticles in a rotating homogeneous field can be calculated via the phenomenological relaxation model of Debye^37^ in the Brownian motion regime:

2 $$\mathrm{tan}\alpha =\omega \frac{2{\tau }_{B}}{2+\xi \bullet L\left(\xi \right)}$$

with $\xi =\frac{mH}{{k}_{B}T}$ and the Langevin function $L\left(\xi \right)=\mathrm{coth}\xi -{\xi }^{-1}$, whereas m = M/V is the magnetic moment of a single nanoparticle of volume V, k_B_ is the Boltzmann constant, and T is the temperature. The Brownian relaxation time is given by ${\tau }_{B}=\frac{3\eta {V}_{hydro}}{{k}_{B}T}$ (η: viscosity of fluid).

**Figure 2:**
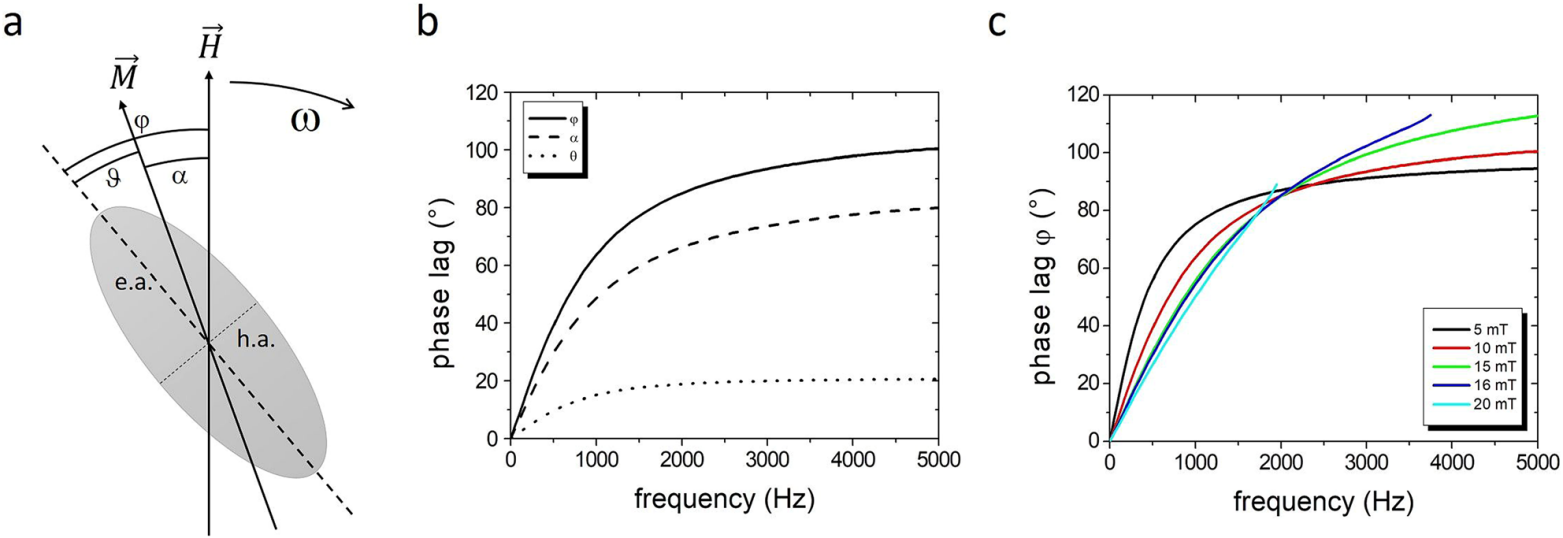
(**a**) Sketch of a rotating nanoparticle with relevant angles. (**b**) Calculated phase lag φ (measured), α (viscous) and ϑ from Eqs. (2) and (4) for a 400 nm x 200 nm elliptical nanoparticle with a NiFe layer of 7 nm and a total thickness of 60 nm in water (η = 0.001 Pa s) at room temperature for H = 10 mT. The saturation moment of NiFe is 88 emu/g, the anisotropy field 30 mT, and the hydrodynamic dimensions are increased by a factor 3. (**c**) Optically measurable phase lag φ for different field strengths.

The optically observed phase lag φ, referencing to the rotating field, typically coincides with a principal axis, e.g., long axis, of an elliptical nanoparticle, which only equals to the angle α of the particle’s magnetization direction in the case of a rigid dipole moment along this axis. This, however, is usually not the case. The magnetization behavior in a ferromagnetic elliptical-cylindrical nanoparticle is approximated by the single-domain Stoner-Wohlfarth (SW) theory^38^. Figure 2a illustrates the situation of a flat ellipsis under viscous rotational drag. The magnetic energy is given by

3 $$E={E}_{0}+K{\mathrm{sin}}^{2}\vartheta -{\mu }_{0}HM\mathrm{cos}\left(\varphi -\vartheta \right)$$

in the presence of shape anisotropy with $K=2\pi {M}^{2}\left({N}_{long}-{N}_{short}\right)$ and the Zeeman term. N_long_, N_short_ are the demagnetizing factors along the long and short axis. The equilibrium state is reached if the first derivative is zero, which results in

4 $$\mathrm{sin}\left(2\vartheta \right)=2\frac{H}{{H}_{K}}\mathrm{sin}\left(\varphi -\vartheta \right)=2\frac{H}{{H}_{K}}\mathrm{sin}\left(\alpha \right)$$

with the anisotropy field ${H}_{K}=2K/{\mu }_{0}M$ and the angle relation $\varphi =\vartheta +\alpha$ (Fig. 2). Thus, the measured phase lag φ is calculated by Eqs. (2) and (4). As shown in Fig. 2b, the viscous lag α and the optical observable phase lag φ increases with the angular velocity ω. A low magnetic field H causes a small magnetic torque Γ leading to a large viscous phase lag α and, accordingly, a large rotational phase lag φ at low rotational frequencies (Fig. 2c). The application of higher fields shifts the phase lag to higher frequencies due to a stronger magnetic torque. Another phenomenon is the increase of ϑ with the field H, which finally reaches the magnetic instability point on the SW asteroid under the conditions $H/{H}_{K}\mathrm{sin}\varphi ={\mathit{sin}}^{3}\vartheta$ and $H/{H}_{K}\mathrm{cos}\varphi =-{\mathit{cos}}^{3}\vartheta$. Consequently, the enforced magnetic switch can lead to chaotic system of the nanoparticle in fluid. This SW description is an approximation for fully magnetized nanoparticles. The instability is already reached at smaller fields if the nanoparticles are not saturated, e.g., in the multi-domain state.

The assay concept additionally requires optically anisotropic nanoparticles, which allow the observation of the average nanoparticle orientation in the analyte solution by linearly polarized light. The optical polarizability of anisotropic nanoparticles represents a non-scalar quantity and leads to a different scattering behavior depending on the relative orientation of the particle’s principal axis to the direction of the polarization plane given by its electric field vector^39^. Thus, particles with an optical anisotropy would already be sufficient for observing the phase lag signal. However, plasmonic resonances additionally enhances the scattering or extinction cross-section by a factor of several hundred, at best. This enables the use of lower particle concentrations in the analyte sample, which leads to a higher target-molecule-to-nanoparticle ratio and, thus, significantly improves the limit of detection. Hence, ideal anisotropic nanoparticles are both magnetic- and plasmonic-active.

### Top-down fabrication of multifunctional nanoparticles

The top-down approach for the fabrication of nanoparticles is motivated by the complex tasks and requirements of homogeneous immunoassays. While simple passive nanoparticles suffice for signaling in most heterogeneous immunoassays, homogeneous assays often require nanoparticles equipped with several functionalities at the same time. In this case, they need magnetic activity for agitation by an external magnetic field, excellent optical responsivity to detect their orientation in the solution and, of course, surface functionality for specific binding of the target molecules. Appropriate magnetic response and plasmonic response at a certain wavelength is adjusted by adequate size and material choice. Both the magnetic and plasmonic property variation should be kept as low as possible, which requires a very narrow size distribution. This precision also results in a uniform rotation in the analyte solvent. Any size distribution would immediately lead to a phase lag distribution, and hence to sensitivity loss. Chemically synthesized nanoparticles are not able to fulfill all these requirements with accurate precision. It is yet complicated to synthesize a narrow size distribution of nano-objects from a single (magnetic) material. Core/shell nanoparticles with a magnetic core and a plasmonic shell have been tried to synthesize, but the procedure is rather complex and the achievable homogeneity in size distributions is limited^40^. Due to surface energy reasons in equilibrium growth, island-growth is preferred in thin films, such that the shell (e.g., Au on Co) is non-continuous leading to arbitrary plasmonic effects. By using thin film deposition techniques such as thermal evaporation or sputter deposition, proper non-equilibrium growth can be achieved. Performing nanoimprint lithography and sputter deposition to create hybrid nanoparticles is an advanced way of combining different materials’ properties in nearly any possible particle shape. The aim was to create nanomaterials exhibiting very narrow polydispersity indices and uniform physical, chemical and magnetic properties.

A high degree of magnetic anisotropy is necessary for the particles to exhibit an optimum rotational drag torque in an external magnetic field. The plasmonic peak wavelength should be adjusted as well and positioned between 700 and 1100 nm because water, serum and blood have here an optical transmission window with less absorption^41^. Optical anisotropy and a high degree of uniformity of the nanoparticles are necessary to ensure assay sensitivity. Functionalization of the particle surface, for example using gold-thiol crosslinking, enables the binding of target molecules. In order to maximize the relative increase of the hydrodynamic drag upon addition of antigen/protein, a small nanoparticle size is favored. Smaller particles, however, have lower magnetic moments and extinction cross-sections. Further, there are limitations in particle size as NIL fabrication reliability, reproducibility, and therefore monodispersity are deteriorated below about 100 nm. An optimization needs a compromise. NIL provides an inexpensive technique to fabricate nanoparticles with high throughput by using stamps with areas of several cm^2^. The required number of nanoparticles is estimated in the Supplementary Materials. The final release of the nanoparticles into solution demands to develop specific NIL processing since nanoimprint materials and processing have to be adapted to the lift-off and solution chemistry.

### Nanoimprint lithography, thin film deposition and lift-off

**Figure 3:**
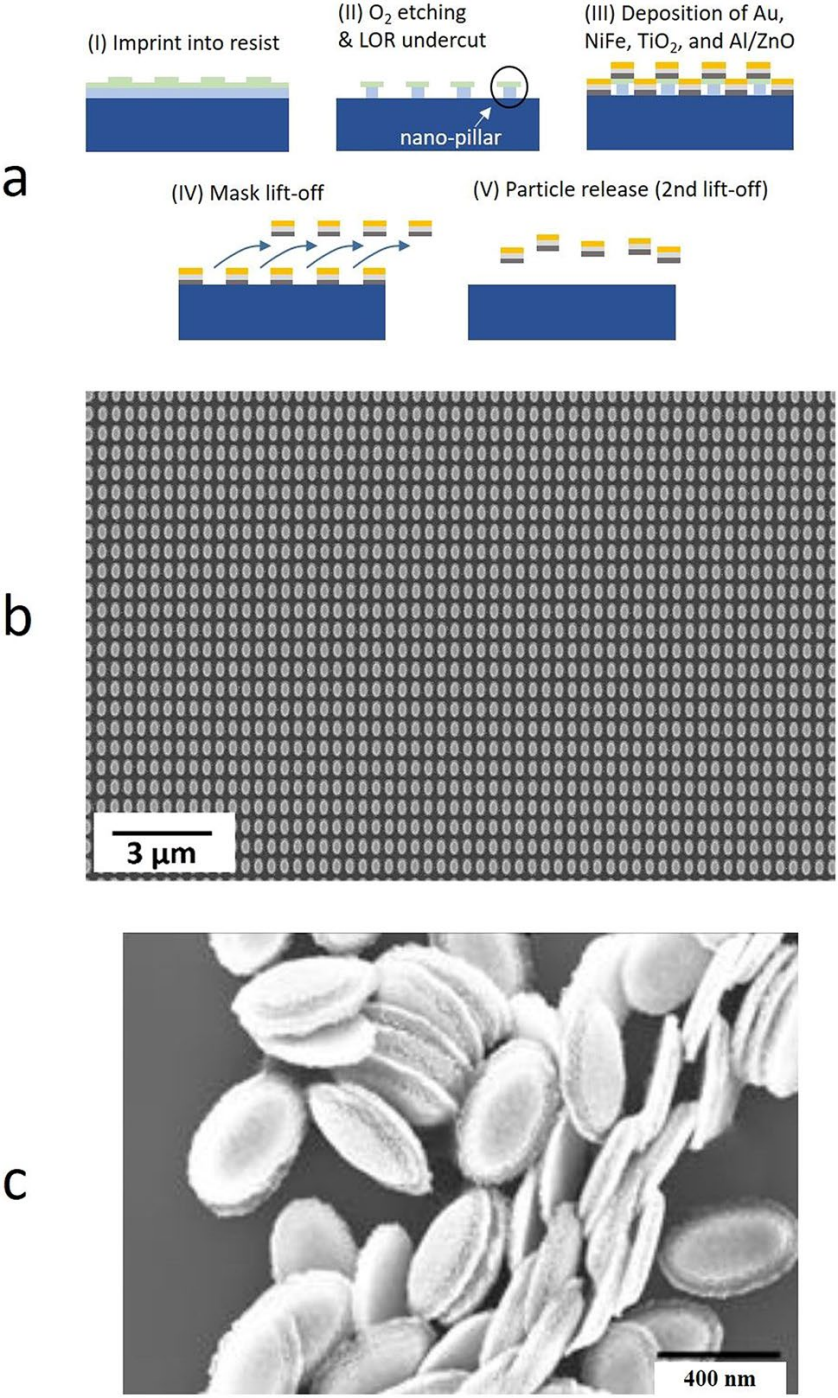
(**a**) Schematic illustration of the NIL fabrication steps. (**b**) SEM image after sputter deposition and mask lift-off (step IV). (**c**) SEM micrograph of released nanoparticles after the lift-off process (step V).

The top-down fabrication of releasable nanoparticles is an important step (Fig. 3a) and shortly summarized in the Methods below. More details of the NIL procedure itself can be found in^27^, and an imprint with an inverse structure for the fabrication of nanoparticles with hollow pockets was reported previously ^42^. A typical nanoparticle nominally has an elliptical-cylindrical shape of 200 nm × 400 nm axes lengths and a sputtered material stack consisting of a combination of Gold (Au) as plasmonic/functionalizing layer, Permalloy (Ni_80_Fe_20_ = Py) as magnetic component, Titanium-Dioxide (TiO_2_) as dielectric spacer layer, and Al/ZnO as sacrificial layer for release.

The quality and homogeneity of the nanoparticles on a large-scale is decisive for their usability as multifunctional nanoprobes. Figure 3b shows SEM micrographs of nanoparticle arrays on substrate. The lateral size variation is evaluated to + -3% and Gaussian distributed ^43^. Dissolved and dried, the nanoparticles exhibit a uniform shape with a small brim on the side, which consists of material remnants from the preparation process (Fig. 3c). This brim obviously plays a minor role for the investigated properties.

### Magnetic properties

**Figure 4:**
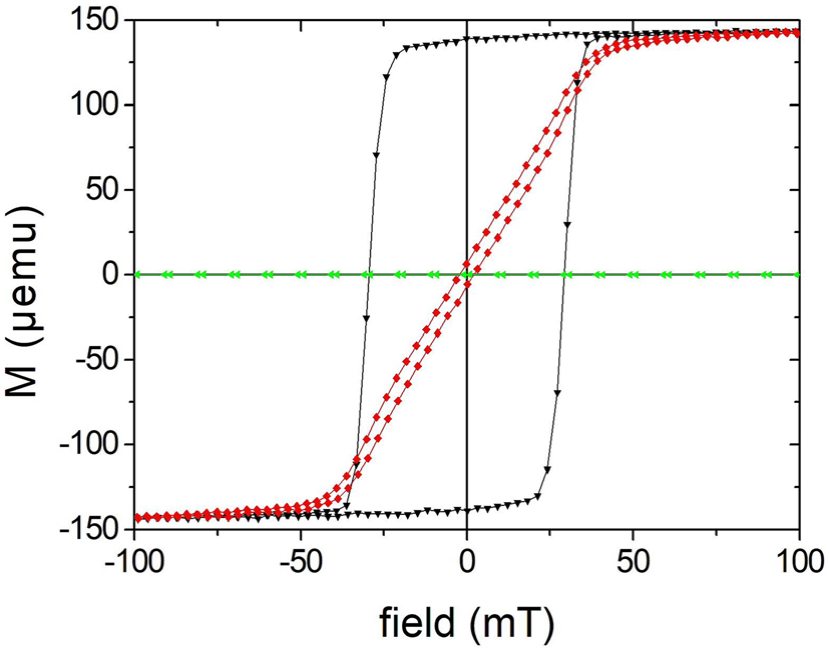
Magnetic hysteresis curve of an ellipses array with 7 nm NiFe thickness with field in-plane along the long (black)/short (red) axis and out-of-plane (green).

Though still not perfect (e.g., brim), the nanoparticles exhibit a uniform magnetic behavior. Figure 4 shows the hysteresis curves along the long axis (easy magnetization axis) and the short axis (hard magnetization axis) for a complete ellipses array, which still resides on the substrate (as prepared, not dissolved). The softmagnetic NiFe with a layer thickness of 7 nm is protected by TiO_2_ layers against oxidation. The steep transition at the coercive field H_c_ = 25 mT with a transition width of only 1.7 mT (10% to 90% of saturation) results from the narrow size distribution since H_c_ essentially depends on shape anisotropy. The anisotropy field H_k_, which indicates the saturation field in the hard axis, equals about 30 mT.

The magnetic anisotropy and a remanent state in the long axis are responsible for a stable in-plane rotation of the nanoprobes as explained in the previous chapters. Since the out-of-plane moment (green points in Fig. 4) of the nanoparticles can be neglected due to the shape anisotropy in thin films, which forces the moment into the plane, the nanoparticles are stabilized in-plane in fluid. The larger the differences of the torque values in easy and hard axis, which is the case at small fields, the more stabilized is the nanoparticle. Pre-requirement, however, is an initial magnetizing of the nanoparticles to saturation in order to set the full moment. The drawback of a permanent magnetization is a possible stray field interaction and agglomeration of the nanoparticles in solution, but this can be balanced by electrostatic, steric or hydration interactions^24,44^.

In conclusion, the 7 nm thick NiFe nanoparticles show a characteristic behavior of a quasi-single-domain state. The description in the SW framework and Eqs. (3) and (4) are therefore justified and a good approximation. A thinner layer leads to a lower moment and, thereby, lower torque, which deteriorates the rotation stability. Thicker NiFe ellipses (> 20 nm) exhibit a magnetic vortex state (not discussed here). The magnetic anisotropy also plays a major role because it effects a stable rotation. A circular shape without any in-plane anisotropy is unable to produce any rotational torque. An increase of anisotropy due to, e.g., an elongation of the ellipsis favors a torque stability but is limited by the fact that the magnetic configuration transits from the quasi-single-domain state to a multi-domain situation during switching at a certain ratio of long/short axis^45^. Such a state generally reduces the remanence. Micromagnetic simulations indicate an optimum ratio of long/short axis between 2 and 3.

### Experimental data and numeric modelling of the plasmon-optical properties

**Figure 5:**
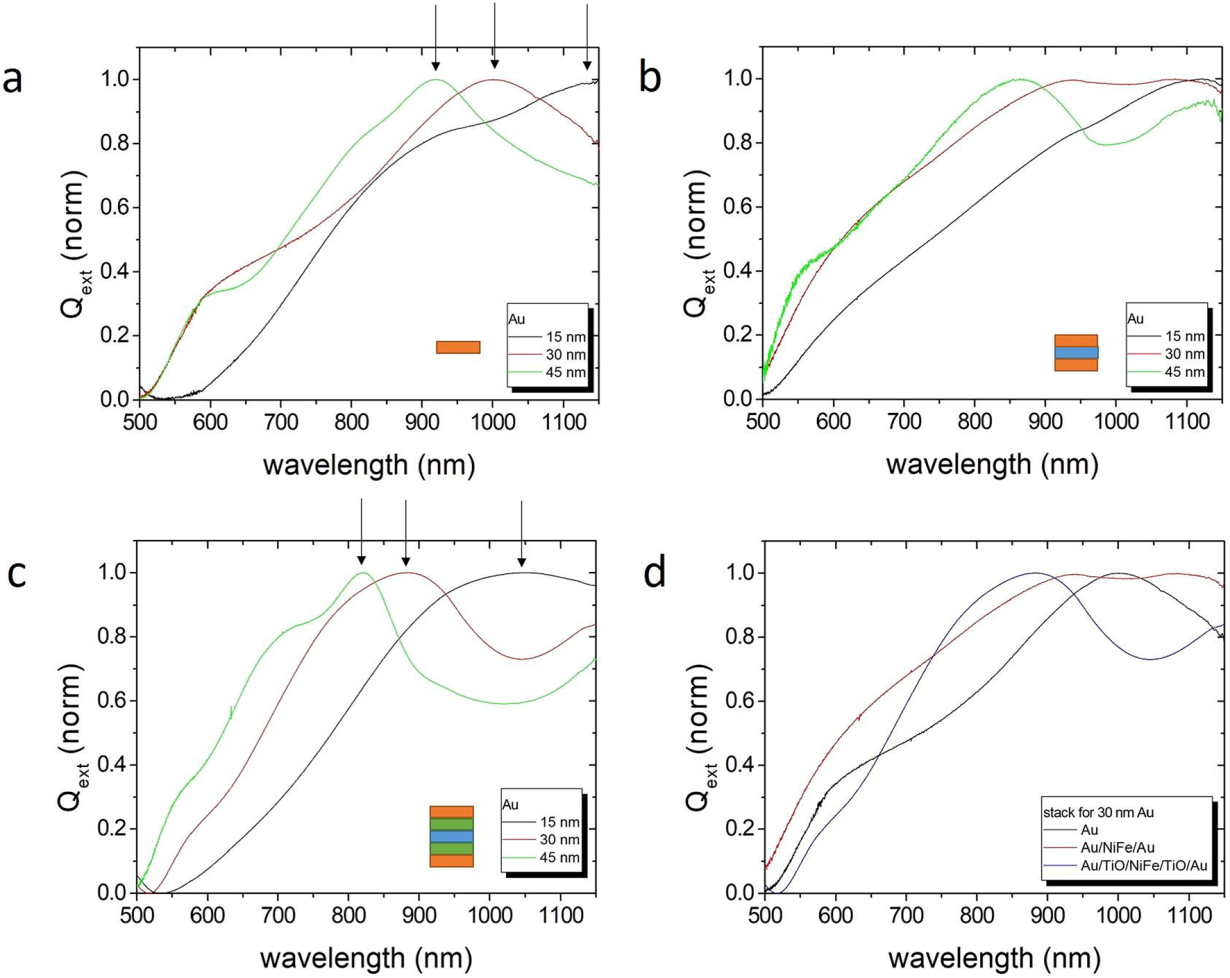
Experimental normalized extinction factors Q_ext_ for different material stacks and Au layer thicknesses (15, 30, and 45 nm) in solution, i.e. with random particle orientation. (**a**) Single Au layer, (**b**) stack without spacer layer: Au/NiFe 10 nm/Au, (**c**) complete stack: Au/TiO_2_ 10 nm/NiFe 10 nm/TiO_2_ 10 nm/Au, (**d**) comparison of stacks for 30 nm Au. Absorption maxima are indicated by arrows.

**Figure 6:**
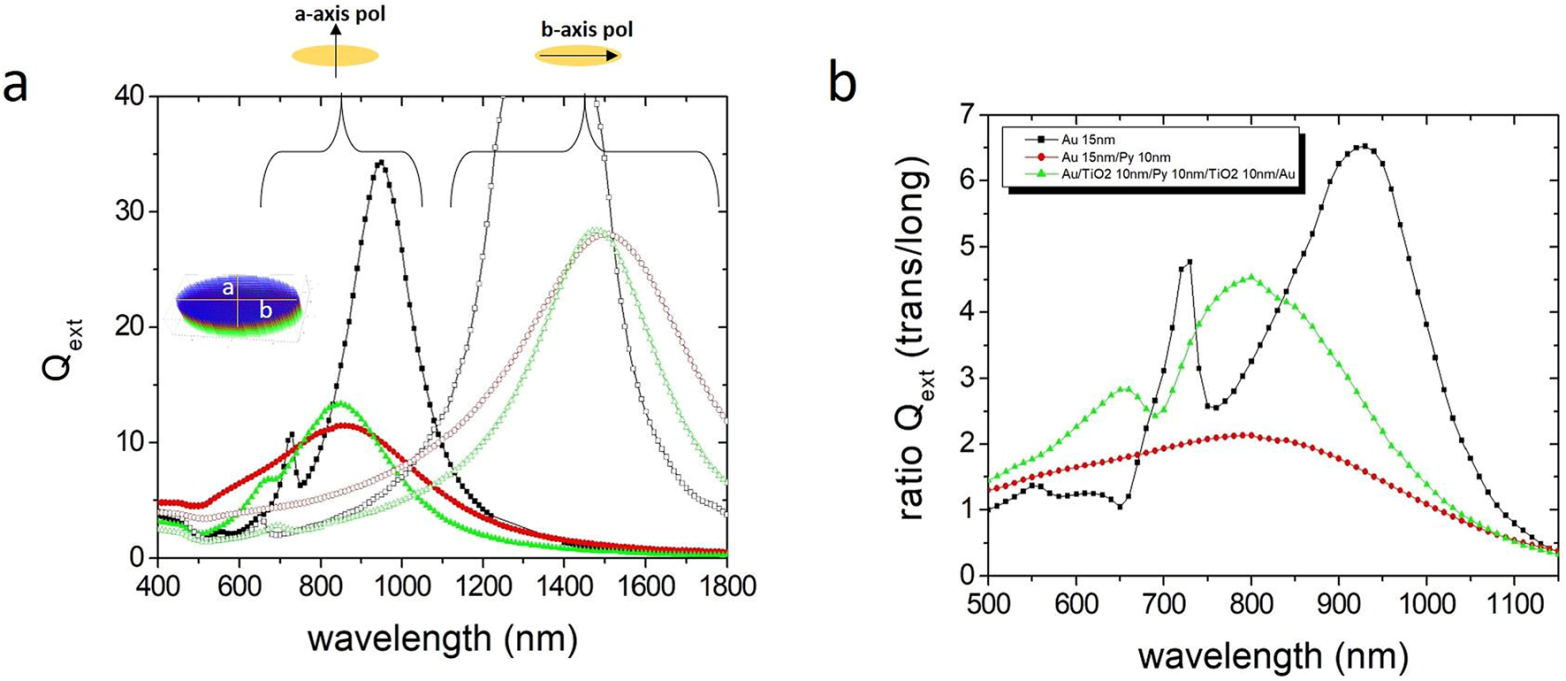
Simulated extinction factors Q_ext_ for different ellipses stacks. (**a**) Q_ext_ for light polarization parallel to the short and long axis as indicated by the inserts, for only Au (black), Au/Py (red), and Au/TiO_2_/Py/TiO_2_/Au (green). (**b**) The ratio of transversal/longitudinal extinction factors Q_ext_. The five-layer stack shows a pronounced peak at 800 nm.

A direct comparison of experimental data (Fig. 5) and numerical simulations (Fig. 6) reveals the presence of plasmon modes in multilayered elliptical nanoparticles and the ability to tailor the resonance peaks by a proper layer composition and thickness choice. Both the experiment and the simulation are described in “Methods” section. In order to understand the plasmonic behavior of the complex multilayered nanoparticles, a systematic investigation in dependence of the Au layer thickness and stack composition is presented in the following. The focus is on 200 nm × 400 nm ellipses with different stacks and Au thicknesses.

In the DDA simulations, the polarization direction of the linearly polarized incident light is oriented either parallel to the long or short ellipses’ axis leading to a longitudinal or transversal plasmon mode excitation, respectively. The measured detector signal yield is proportional to the difference in extinction between these two excitation modes, with a maximum, when only one of the plasmonic resonances lies within the detection window. The simulation calculates efficiency factors ${Q}_{x}\equiv {C}_{x}/\pi {a}_{eff}^{2}$ (dimensionless number), with ${C}_{x}$ representing either the scattering, absorption or extinction cross-section (area units), while ${a}_{eff}$ is a mean effective particle radius. Important for the sensitivity is the relative ratio of the extinction cross-section Q_ext_ of longitudinal and transversal plasmon mode at the measurement frequency. This ratio directs the measurable optical signal amplitude of the rotating nanoparticle (cf. Figure 1b).

Experiments with single Au layer ellipses prove the development of a plasmon mode and a blue-shift of the plasmon peak at 1148, 1000 and 920 nm wavelength with increasing Au thickness of 15, 30 and 45 nm (Fig. 5a). The curves are normalized because the nanoparticle concentration varied in the experiments. Such a plasmon resonance is also seen in the simulations (Fig. 6). A 15 nm thick simulated Au nanoparticle exhibits a transversal plasmon peak at 950 nm wavelength, which shifts to 825 nm and 760 nm for 30 nm and 45 nm Au thickness (see Supplementary Materials). Although the experimental peaks are shifted in wavelength, which may result from roughness, deformation or impurities, it can be deduced that the plasmon peak in the experiment at wavelength < 1 µm is generated by the short axis. Plasmon resonance peaks in the near infrared at wavelength >  > 1 µm are caused by the long axis.

The vicinity of another metal, like NiFe in this case, strongly suppresses the transversal plasmon peak due to quenching (Fig. 5b). The simulations confirm this behavior. The peak of the Au/Py double layer decreases and broadens compared to the single Au layer. The maximum shifts to smaller wavelength. The longitudinal peak shifts opposite to the infrared. The simulations show that the insertion of a dielectric spacer layer like TiO_2_ again narrows the peak widths and blocks the quenching to some extent (see Supplementary Materials). Taking advantages of a spacer layer and building symmetric nanoprobes with a 5-layer stack like Au/TiO_2_ 10 nm/Py 10 nm/TiO_2_ 10 nm/Au with Au on both sides for functionalization (e.g., thiol reaction), the results improve substantially (Fig. 5c and Fig. 5d). The transversal plasmon peaks at wavelengths of 1050, 880, and 820 nm become nicely pronounced with a narrow width. The simulation reproduces these features (green curves in Fig. 6).

The ratio of the extinction factor amplitude Q_ext_ of the transversal and longitudinal plasmon correlates with the sensitivity and is calculated from the simulations (Fig. 6b). While a single Au layer would have the largest ratio of about 7 and, hence, largest sensitivity, the necessary magnetic layer quenches the ratio by almost a factor 3. The 5-layer stack with dielectric separation layers regains a ratio of 4.5 resulting in a reasonable sensitivity, similar to the experimental data.

### Protein detection by imprinted nanoprobes

**Figure 7:**
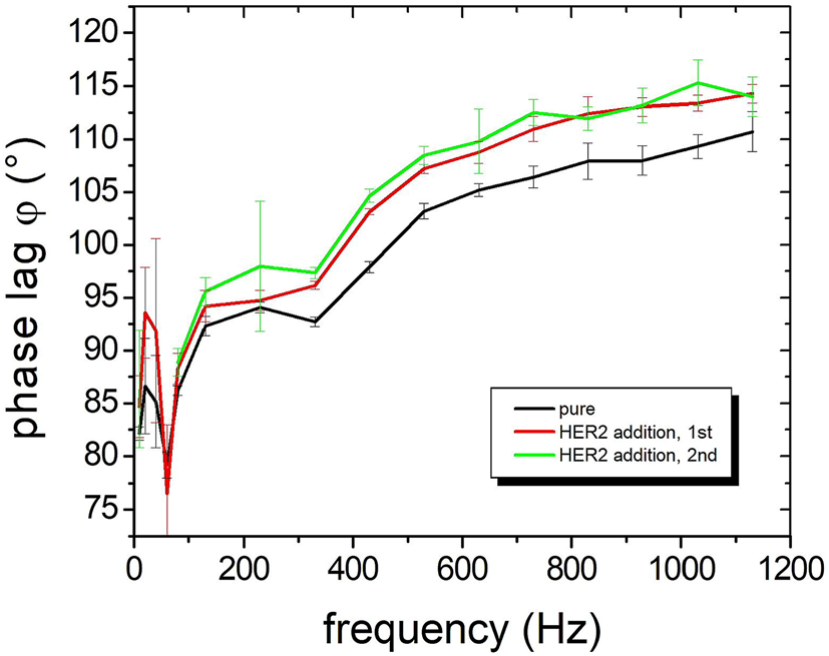
Demonstration of the phase lag increase of 5-layer nanoprobes functionalized by sHER2 antibodies on addition of sHER2 proteins (sHER2 assay). The nanoparticle concentration is 10 pM and the oscillating magnetic field amplitude is 5 mT. The binding sHER2 proteins are added twice.

The rotational dynamics of imprinted nanoparticles in fluidic environment have been investigated in dependence of the rotating magnetic field. Inferring from the last chapters, a magnetic orientation and rotation approximating the long axis in small fields and a plasmonic excitation along the short axis at the laser wavelength of 830 nm is expected. This means that the optical signal will always be shifted by 90° relative to the particle’s magnetization, the latter of which will be approximately along the long axis (i.e., α ~ φ, compare to Fig. 2a). As a result, optical signals with a phase lag relative to the magnetic field approaching 90° are expected for low frequencies of the oscillating field, which then will increase above 90° as the frequency rises. In Fig. 7, this 90° shift is measured at low frequencies. A steady increase of the rotational frequency leads to a nonlinear increase of the phase lag due to the viscosity forces in the buffer, with a tendency in reaching a saturation level at higher frequencies similar to the calculated curves in Fig. 2. These measurements at a field of 5 mT are performed with the 5-layered nanoprobes in PBS buffer solution. The nanoprobes are functionalized by sHER2 antibodies. Assays with HER2 are relevant in breast cancer therapy. By adding sHER2 proteins at a concentration of 10 nM into the buffer, they successively attach to the nanoprobes, which leads to a further increase in phase lag. The significant phase lag variation at low rotational frequencies (negative peak below 100 Hz) is reproducible, but not explicable yet. Dynamical respectively rotational effects of the asymmetric bodies in the fluid might be the origin.

The specific binding to target molecules is a necessary prerequisite for a successful immunoassay. Two key factors are a high binding affinity of the probe to the target molecule and secondly a high-quality surface functionalization of the nanoprobes by the biological probes. While the first factor depends on biology and biomolecular engineering, the latter is a task of proper and clean chemistry. A successful implementation in real samples of serum and saliva has been shown with the detection of the breast cancer biomarker sHER2 by functionalized rotational nanorods^25^.

**Figure 8:**
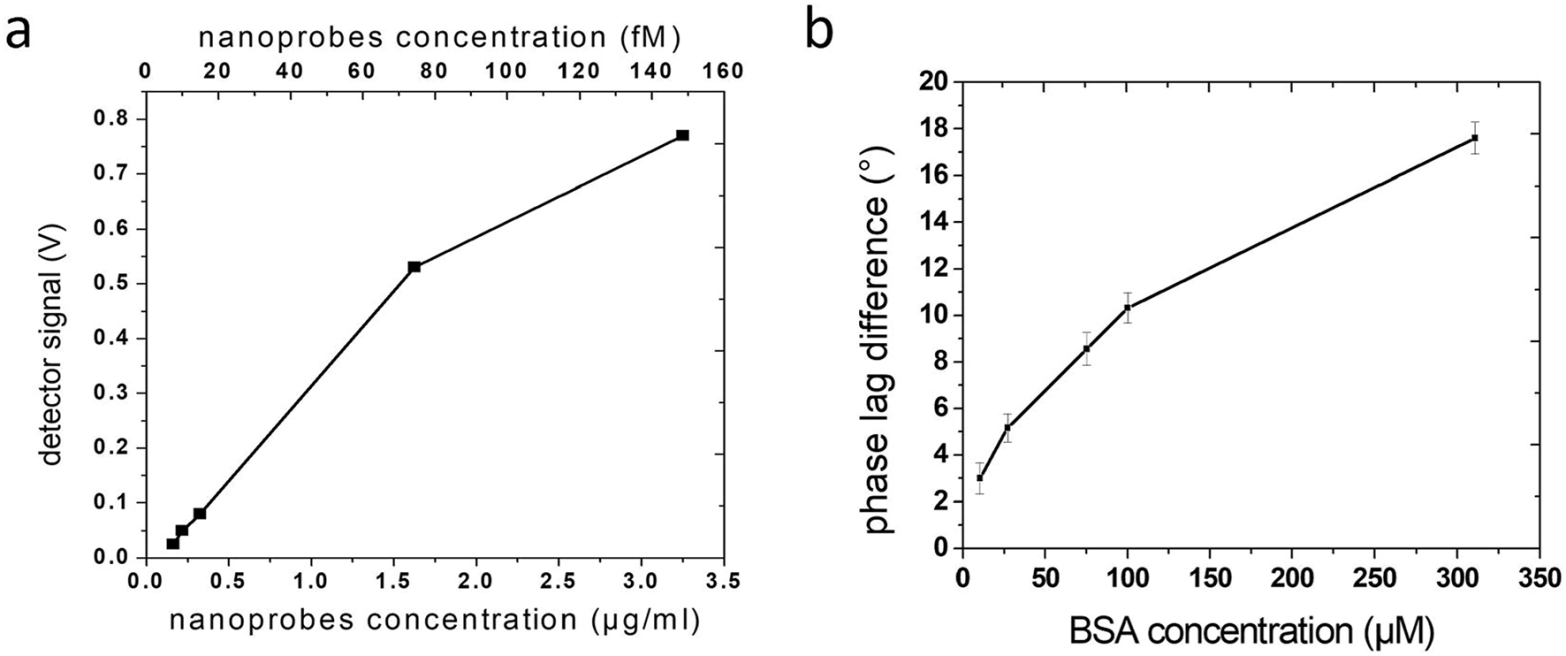
(**a**) Detector signal vs. nanoprobes concentration for a field amplitude of 5 mT. (**b**) Phase lag difference signal at varying BSA concentrations added to 230 ng/ml nanoprobes.

In a second test, Bovine serum albumin (BSA) serves as an inexpensive model protein for disease-related biomarkers or pathogens and is applied to study changes in the rotational dynamics of nanoparticles upon biomolecular binding events. All biomolecular detection measurements are carried out in ultrapure water with 5-layer-stacked nanoprobes. The surface area is not bio-functionalized. The detected signal, which is proportional to Q_ext_, depends on the amount of nanoprobes in the solution. The signal increases linearly with the nanoprobes concentration and starts to saturate at large concentrations (Fig. 8a). For the detection experiments with BSA, a nanoparticle concentration of 10 fM is chosen, which clearly exceed the detection limit and guarantee a stable signal. The BSA protein and the nanoprobes are dissolved in ultrapure water and homogenously mixed before running the measurement. By comparing the phase lag of the reference with the BSA-nanoparticle measurements, the phase lag difference is determined, thus indicating the amount of bound BSA protein. The measurements are carried out at 900 Hz and 5 mT. The phase lag difference decreases if the amount of added BSA is successively reduced (Fig. 8b). In principle, the phase lag also depends on the fluid viscosity (Eq. (1)). The effect of BSA protein addition to an aqueous solution on the viscosity have been investigated, and no measurable increase of the viscosity have been observed at BSA concentrations up to 1 µM^46^. Up to 4% variation in viscosity can be measured at concentrations of 100 µM. This variation corresponds to 2% variation in phase lag, which is smaller than the error bars of standard deviations in Fig. 8b. The analysis of the absolute detectivity and sensitivity range is subject to further investigations.

For future work, there are several strategies to improve the sensitivity. An increase of the ratio of the extinction factor amplitudes Q_ext_ directly yields a higher sensitivity due to an easier detection ability. This can be achieved by further tailoring the plasmonic response via material and geometry optimization or by shifting the wavelength to the infrared. For example, it can be estimated from Fig. 6a that Q_ext_ ratio of the 5-stacked nanoprobes is 44 at a wavelength of 1470 nm, which is a factor 10 larger than at 800 nm. While the magnetic part is already fine, a long light path through the sample would increase the total cross-section. A possibility could be the utilization of multiple reflections in a cavity, for example. This could deliver another factor of 10 in sensitivity gain. A decrease in nanoprobe size would also lower the limit-of-detection by the fact that a smaller base volume yields a relative larger volume difference by adding material leading to a larger phase lag difference.

## Conclusion

Multicomponent nanoparticles fabricated by a top-down approach of thin film deposition and nanoimprint lithography are highly monodisperse in size as well as in magnetic and plasmonic properties. The plasmon-optical properties are well reproduced by numerical simulations and can be tailored to the requirements imposed by the nanoimprint process, analyte sample, and the detection principle. Magnetic properties can independently be adjusted, as well. The optically measured phase lag is different from the viscous phase lag, which is explained in the framework of SW theory and can lead to rotational instability in high driving fields. Rotational dynamics experiments reveal a lowest detectable particle concentration in the fM regime. The biomolecular detection limit lies in the lower µM regime. The imprinted nanoprobes provide a tailorable versatility, which can be advantageous in other applications, too.

While an in-vitro application of multifunctional nanoprobes as immunoassay is possible with a reasonable operating expense, any in-vivo application for, e.g., diagnostics and therapeutics, complicates the situation. While smaller extremities like fingers can be easily supplied with a rotating magnetic field, a whole body requires big excitation coils of enormous size. In addition, the optical detection scheme is limited to the penetration depth in human tissues. Even if the detection is shifted to the infrared, the typical range is only a few millimeters^47^.

## Methods

### Fabrication processes of the nanoprobes

A flexible working stamp is fabricated from a master mold comprising a 1 × 1 cm^2^ array of 200 nm × 400 nm elliptically shaped pillars of 250 nm height using an h-PDMS/PDMS hybrid stamp. The higher modulus h-PDMS (“hard-PDMS”) contains the nanopattern and guarantees a high-resolution replication while the low elastic modulus Sylgard 184 PDMS enables the flexibility of the hybrid stamp. In order to produce a flexible working stamp, the h-PDMS is spin-coated on the master and pre-cured for 10 min. After the h-PDMS, a 1 mm thick PDMS layer is deposited onto the h-PDMS and then cured over night at 60 °C. For imprinting, a double-layer system including a LOR1A lift-off resist and mr-NIL212FC imprint resist (microresist technology GmbH) is used. LOR1A is spin coated on a Si-wafer with 4000 rpm to a layer thickness of 100 nm, followed by a 5 min hard bake at 100 °C on a hotplate. Afterwards, the imprint resist mr-NIL212FC is spin coated with 4000 rpm followed by a 1 min soft bake at 100 °C. For the imprinting step, the stamp is pressed into the coated Si/SiO_2_-wafer for 30 s to ensure a complete filling of the stamp nanopattern, and afterwards cured for 1 min with a high-power UV LED (intensity = 100 mW/cm^2^, λ = 365 nm). After the separation by peeling the stamp off, an anisotropic oxygen plasma etch removes the residual layer of the imprint and etches through the LOR1A resist. This is followed by a developing step in diluted MICROPOSIT MF-24A developer for 30 s to selectively etch the LOR1A and thus create an undercut. Once the imprinting is completed, the plasmonic (Au), magnetic (Ni_80_Fe_20_), passivation (TiO_2_), and bottom sacrificial layer (Al-doped ZnO) layers are sputtered (UNIVEX 450C from Leybold Systems, Cologne). With the help of the undercut in LOR1A, the mask can be lifted off the substrate by a short immersion in MF-24A (step IV in Fig. 3a), whereas the remnant nanoparticles can be brought into solution through a second immersion in a developer, which etches the bottom sacrificial layer (ZnO). In a final step, the nanoparticles are transferred into ultra-pure water by a three times magnetic separation repetition using conventional permanent magnets.

To further achieve a stable nanoparticle dispersion and to allow for antibody protein binding to the nanoparticle surface, a surface modification of the nanoparticles with a heterobifunctional crosslinker was carried out. To that end, a polyethylene glycol (PEG) polymer with a thiol moiety on one end to facilitate the binding of the crosslinker to the gold surface and a carboxy group on the other were employed. In aqueous solution, the carboxy functionality is necessary to allow for electrostatic nanoparticle dispersion stabilization and to further perform EDC-S-NHS (1-Ethyl-3-(3-Dimethylaminopropyl) Carbodiimide (EDC) and Sulfo-N-Hydroxysucchinimide (S-NHS)) coupling between the carboxy group on the crosslinker and amino moieties on the antibodies.

The surface modification is presented in detail in the following. First, the sample after the mask lift-off was cleaned by a 1:5 dilution of piranha solution for 5 min to remove all remaining resist residues. Second, the sample was placed in an aqueous solution of the PEG crosslinker and placed overnight on a platform shaker (10 kDa PEG with a concentration of 10 molecules per nm^2^ of the nanoparticle surface). This step already coats the top surface of the nanoparticles with the crosslinker. In a third step the particles were lifted-off the substrate by immersion into a solution of 0.1 M NaOH, which dissolves the sacrificial AZO layer. The latter process was executed in an ultrasonic bath for 1 h. Additionally, the crosslinker PEG molecule was added to the NaOH solution to allow for coating of the bottom nanoparticle surface during the lift-off process (again with a concentration of 10 molecules per nm^2^ of the nanoparticle surface). After a magnetic washing step to remove all non-magnetic material from the sample, an aqueous solution of the same crosslinker was added and the sample transferred to Eppendorf tubes, which were placed in an overhead shaker overnight. After this process, the gold surface is coated by the crosslinker. The particles were then washed by repeated magnetic washing and transferred to ultra-pure water or phosphate buffered saline (PBS) at pH 7.4. In a final step, the nanoparticle surface was modified by antibodies, which was achieved by EDC / S-NHS coupling chemistry. EDC and S-NHS were added in PBS solution at concentrations of 1 × 10^5^ and 3 × 10^5^ per nm^2^ of the nanoparticle surface and the mixture incubated for 20 min at room temperature. Then, the antibodies were added at a concentration of one antibody per nm^2^ of the nanoparticle surface and the sample first incubated for 90 min at room temperature and then stored in the fridge overnight. Finally, the functionalized nanoparticle dispersion was washed by magnetic separation in PBS solution.

### FTIR spectroscopy

The optical data are generated from nanoparticles in ultrapure water in transmission in a FTIR spectrometer (Bruker). The nanoparticles in the solution represent a mixture of all axes directions because no magnetic fields could be applied in the spectrometer. The simulation data (Fig. 6) are split into the axe’s directions, i.e., longitudinal and transversal mode.

### Numeric simulation of the optical-plasmonic properties

A discrete-dipole approximation (DDA) code DDSCAT^48^ is used to study the extinction, scattering and absorption cross-section of the nanoparticles. The complex refractive index values of gold^49^, Permalloy^50^ and titanium-dioxide^51^ were taken from experimental data. The particles are assumed to be suspended in water with a real refractive index of 1.333, which is comparable to the typical analytes, such as serum or blood.

## Supplementary Information

Supplementary Information
