# Supplementary material for: Nanoimprinted multifunctional nanoprobes for a homogeneous immunoassay in a top-down fabrication approach
Source: Sci Rep. 2021 Mar 16;11:6039. doi: 10.1038/s41598-021-85524-8 (PMC7971043; doi:10.1038/s41598-021-85524-8)
Supplement: Supplementary file 1 — Supplementary Information [file 41598_2021_85524_MOESM1_ESM.docx]

## Supplementary Data for

## Nanoimprinted multifunctional nanoprobes for a homogeneous immunoassay in a top-down fabrication approach

Hubert Brueckl, Astrit Shoshi, Stefan Schrittwieser, Barbara Schmid, Pia Schneeweiss, Tina Mitteramskogler, Michael J. Haslinger, Michael Muehlberger, Joerg Schotter

### Estimation of the required number of nanoparticles for a single assay

The required amount of nanoprobes for an assay can be estimated from the targeted probe concentration of 1 pM in a typical microfluidic cavity volume of 1 µl. For a referenced measurement, three cavities are required, resulting in a total number of required nanoprobes of about 2 million. Taking into account additional dead volumes, it can be estimated that around 10 million nanoprobes are required for a single assay test. Assuming a master stamp size of 1 cm² and a targeted nanoparticle size of 200nm x 400nm and an inter-particle distance of 200nm, the quantity of at least 100 million nanoparticles can be fabricated in one batch. Additionally, safety buffer in the number of nanoprobes is needed, especially when taking into account losses during nanoprobe purification processes. Including material, machining and personnel for all processing steps, the costs of physically structured nanoparticles in mass production are expected to be below 1 US$ for one assay.

### Further simulation data of the plasmonic behavior

A 15 nm thick simulated Au nanoparticle exhibits a transversal plasmon peak at 950 nm wavelength which shifts to 825 nm and 760 nm for 30 nm and 45 nm Au thickness (Figure S1a). The simulated longitudinal plasmon peak remains nearly stable at 1360 nm, 1300 nm and 1330 nm for the respective thicknesses.

Figure S1. DDA simulations of extinction factors Q_ext_. (a) Transversal and longitudinal Q_ext_ of single Au layers with different thickness (15, 30, and 45 nm). (b) The extinction factor ratio for different spacer layer thicknesses. Absorption maxima are indicated by arrows.


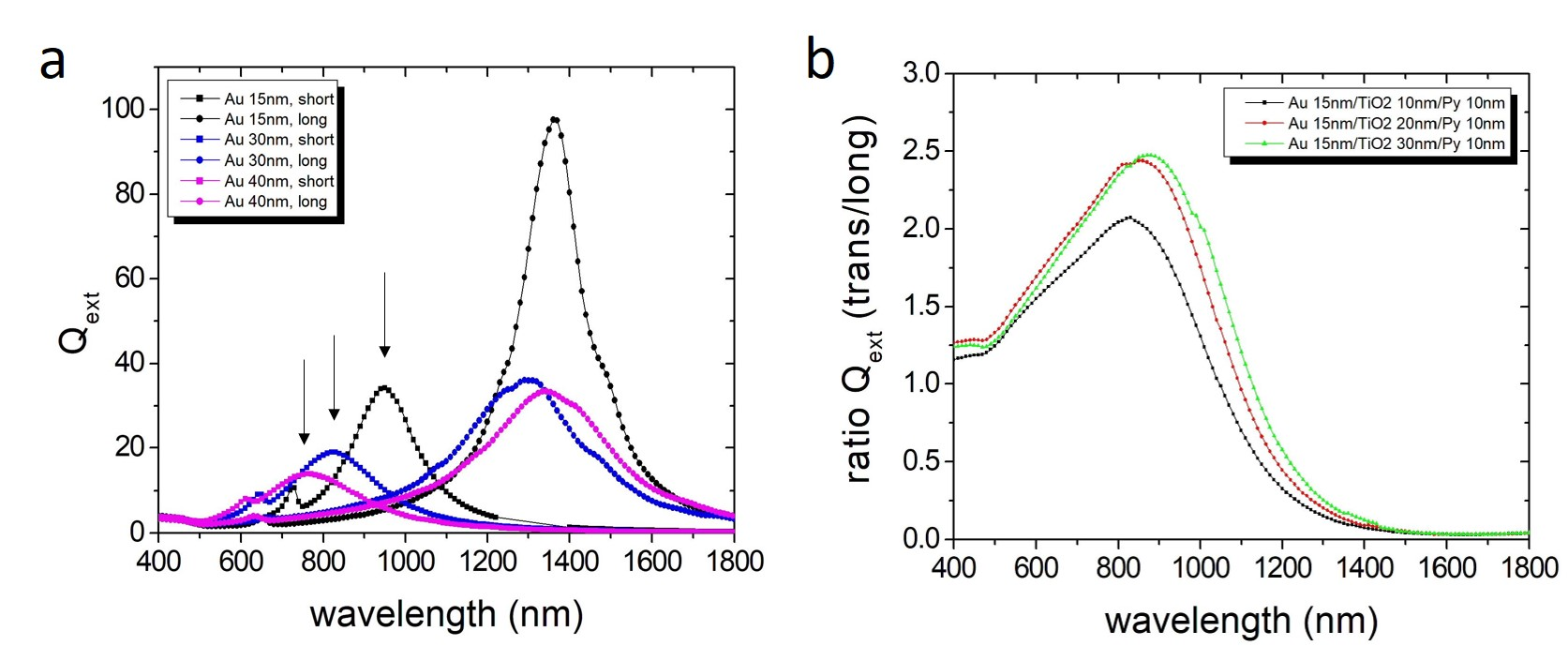


A dielectric spacer layer like TiO_2_ mitigates the Au plasmon quenching by the neighboring metal like Py. An increase of the spacer layer thickness results in an enhanced blocking of the plasmon quenching (Figure S1b) while the peak position is nearly unchanged.
